# Supplementary material for: Basal forebrain degeneration precedes and predicts the cortical spread of Alzheimer's pathology
Source: Nat Commun. 2016 Nov 4;7:13249. doi: 10.1038/ncomms13249 (PMC5097157; doi:10.1038/ncomms13249)
Supplement: Supplementary Information — Supplementary Table 1 [file ncomms13249-s1.pdf]

Supplementary Information

Supplementary Table 1: ADNI participant information

Research ID	Diagnostic Group	Image ID		
		Time 1	***Time 2***	***Time 3***
3	AD	32237	35576	88252
5	HC	32246	31906	200385
10	AD	32270	94368	94377
14	HC	59375	59391	87012
16	HC	32306	31933	200390
21	HC	32332	31970	200399
23	HC	32409	65902	200416
29	AD	119180	82360	133373
30	MC	118750	118793	118836
31	HC	118843	118845	86359
33	MNC	45166	65833	87588
40	HC	34607	47884	87622
41	MC	118697	59854	129868
42	MC	118850	118799	87036
48	HC	45183	68769	89626
50	MC	119270	119271	119273
51	MNC	35819	47889	88309
53	AD	35486	66945	86965
54	MC	35825	47898	87631
57	MC	119796	74240	91468
58	HC	118832	134207	89591

61	HC	119062	118859	87055
66	HC	59446	96110	85934
67	HC	118674	118706	91431
68	HC	118677	35773	86939
72	HC	119274	119276	91932
74	HC	34316	47071	88386
76	AD	35911	63036	133486
77	MC	68120	68125	133465
81	HC	31125	52022	92324
83	AD	31143	52031	92160
84	AD	31205	52044	92169
86	HC	66413	67771	105937
89	HC	49675	72472	94601
90	HC	35841	102436	92337
91	AD	119288	119290	91935
93	AD	31251	62412	91664
94	AD	119300	119306	91942
96	HC	59456	59466	96248
97	HC	64046	60594	108045
101	MC	63297	140762	134657
102	MNC	39460	68176	92012
106	HC	119307	119309	94804
107	MNC	40445	66722	97287
108	MC	119311	75111	165343
109	AD	130231	130233	102511
113	HC	119322	119324	102072

116	MNC	34325	47036	94423
118	HC	34114	47149	96275
120	HC	34332	47023	98785
123	HC	63784	70876	106567
125	HC	35671	67522	91779
127	HC	130234	130236	101739
128	MC	121135	74217	137218
129	AD	59484	59493	94405
130	HC	59524	62702	94414
139	AD	31300	52066	98772
141	MC	33396	67941	98759
142	MNC	118792	118797	99058
147	AD	119079	124892	103333
149	AD	89429	55999	91891
150	MNC	65130	68534	97106
156	HC	39533	68107	91761
158	MNC	45872	66199	102548
159	HC	33489	67958	97039
160	MNC	65739	56004	97182
166	HC	39810	68134	96169
169	MNC	40858	76583	96079
171	HC	65752	68563	97191
172	HC	65757	56013	102790
173	HC	96321	94894	94615
177	HC	34806	64287	133406
179	MC	34141	78876	142362

183	AD	32003	87324	103360
184	HC	40179	83849	102844
186	HC	40201	66418	99122
196	HC	40260	63828	105970
204	MC	39542	66608	99196
214	MC	119086	105747	142010
217	MC	52075	97055	143348
221	AD	72128	102054	104489
222	MC	54686	64764	102450
240	MC	63374	82125	105285
241	MC	32020	35504	200425
249	MC	36530	63306	102648
256	MC	34150	35647	79568
257	HC	34824	74348	133151
259	HC	34362	66190	102516
260	HC	34384	63556	106551
262	HC	124897	87604	103698
269	MC	65257	89486	80424
273	MNC	33428	68448	102711
276	MNC	33433	67986	101425
283	HC	66428	67027	103508
285	MNC	39117	138928	123380
286	AD	118779	118788	108440
291	MNC	34524	65442	101787
292	MNC	39569	67715	102756
295	HC	45108	64025	123685

298	HC	119328	119330	121570
300	AD	40328	66759	107759
301	HC	45881	89089	105942
303	HC	65148	62917	104411
307	MNC	34159	65298	103672
311	HC	39868	80317	135272
316	AD	36573	74627	107975
324	MNC	32891	62599	104357
325	MC	74694	86987	112202
327	HC	79732	72204	108286
328	AD	39620	70661	105878
335	AD	119801	118778	122856
336	MC	34857	64314	133423
337	HC	33437	68006	105303
341	AD	45213	68504	105351
343	AD	33444	85699	105737
344	MC	36579	62621	108040
351	MNC	65401	65620	104372
352	HC	34537	67743	104476
359	HC	96221	78618	121238
361	MNC	59753	64383	105474
366	AD	46608	66206	112391
369	HC	118772	118775	106446
370	AD	59777	87744	106529
372	AD	34549	65451	108067
374	AD	39817	68600	123935

376	MNC	31384	64791	103645
377	MNC	83094	73631	112239
378	MNC	95688	99104	112328
382	HC	59725	80682	109699
384	MNC	48002	80769	105521
386	HC	49680	72499	123913
388	MC	81396	81910	166912
392	AD	59690	72801	109886
394	MC	34398	79391	123776
400	AD	119087	119089	108024
403	HC	34181	70692	105897
404	AD	34204	82214	106485
413	HC	45117	60008	120917
414	MNC	36599	64782	111104
416	HC	39836	94899	107692
419	HC	118701	72162	162073
424	MNC	33644	68032	106451
425	HC	118766	118768	121440
426	AD	40356	66778	111305
429	MC	40387	66806	163740
431	AD	34443	80737	123789
433	HC	39937	72540	106576
434	MC	39122	39190	85589
441	HC	48029	71664	107925
448	MNC	32875	72141	109178
454	HC	79755	71588	108571

457	AD	92406	72919	112366
459	HC	46629	89096	105949
464	MNC	34707	72414	123908
470	AD	34570	88563	108293
472	HC	118702	94946	129183
473	HC	140334	130245	130184
474	AD	34720	64218	109149
479	HC	119090	119092	109517
481	MNC	46647	89103	109968
487	AD	59686	80689	109718
488	HC	107934	89796	109943
497	AD	48038	71669	123416
498	HC	55943	82105	124026
502	HC	51138	87213	123617
507	MC	80199	75462	112547
516	HC	42308	66556	109925
519	HC	39647	71402	123626
520	HC	39660	72170	123635
526	HC	63468	80713	121461
533	HC	38785	68788	112310
534	HC	34578	65464	112319
535	AD	50426	72297	112301
538	HC	40772	78823	163709
539	MC	70710	123142	162775
543	AD	59543	89928	111197
544	MNC	64672	76631	106458

546	MNC	32681	75633	123580
547	AD	75279	78722	123594
552	MNC	79796	88288	112246
553	HC	32644	73520	123585
554	AD	64711	89757	122981
555	HC	39614	102770	165316
557	MNC	39674	71421	123644
558	HC	39683	71434	122818
559	HC	40674	74568	120949
565	AD	39919	75476	123926
567	MC	42370	42375	86686
568	MC	64922	71512	111217
572	MC	32653	73026	122622
575	HC	51160	78805	122796
576	HC	36903	71527	124773
577	AD	36914	72195	108445
578	HC	50459	72306	111320
579	MNC	40410	67015	123962
588	MNC	79824	88302	142027
601	HC	39850	68153	123280
602	HC	32672	74184	122635
604	MC	79191	88040	162265
605	HC	38861	72858	123328
607	MNC	35938	74353	133494
610	HC	32667	74582	122640
618	HC	67110	72188	123017

619	AD	118678	67871	120964
621	MNC	64189	82527	112262
622	HC	34452	72343	123355
625	MC	31495	101445	90880
626	MNC	34672	73903	123847
631	MC	46655	66220	88478
634	MNC	118691	118751	123826
637	HC	118711	118737	122791
638	MC	67531	79053	129842
643	HC	34725	73366	123198
644	MNC	34240	68088	122954
647	HC	33457	71463	123854
648	HC	59666	72814	123289
649	MC	74411	123296	172339
656	MNC	36924	71536	123083
657	HC	59739	82619	123303
658	MC	39701	73041	122843
667	MC	79017	123262	164210
671	MNC	64161	71642	123895
673	MNC	36949	71549	123101
677	HC	119102	119104	123872
679	MNC	40046	74321	123174
680	HC	38926	73200	123337
681	HC	92305	94949	124682
685	HC	40683	74174	120994
686	HC	46668	74550	123971

689	AD	118713	118754	121247
690	AD	50468	73145	123207
692	HC	47295	74367	129580
695	MC	70924	81463	123520
698	MNC	36613	74636	123603
708	MC	72882	123346	162920
711	HC	39137	75651	123253
717	HC	98888	94917	124179
723	MC	42384	51567	96119
724	AD	42400	73959	123024
725	MC	86166	97081	121386
727	MC	63810	105800	124545
729	MC	40708	78654	123994
730	AD	50487	85959	123220
731	HC	90848	78727	124031
733	AD	42425	73989	123033
741	HC	42450	74023	123042
746	MNC	63122	92260	136211
748	MNC	36959	74712	123110
750	MC	59561	99224	122945
752	MC	59637	74419	123310
753	AD	33461	78869	134624
754	AD	80760	80908	124297
759	AD	36969	82336	123119
760	AD	38652	79913	124258
767	HC	47306	124940	124344

768	HC	50506	80276	123225
771	MNC	92286	91189	124335
778	HC	91135	81964	123376
782	MNC	40717	87283	124718
783	MNC	39152	107916	123407
784	AD	39005	82633	163718
786	AD	118990	78770	162100
793	AD	50524	82351	132424
796	AD	89979	91210	137267
800	MNC	43035	79092	123506
803	AD	118716	82120	124069
810	HC	47314	107733	174885
813	HC	36979	79922	124267
814	AD	74591	78700	122649
818	HC	121435	80221	124138
824	HC	96284	81946	122972
829	AD	118707	101977	137223
834	MC	59798	92595	124794
835	MC	78885	122963	162368
839	MC	80230	124525	166957
844	AD	34471	82642	129620
845	HC	64867	78903	124467
850	AD	48996	94797	124458
856	MC	89408	67088	101371
861	MC	67918	101498	162131
866	HC	65611	82274	125011

869	MC	85519	129322	163163
872	MNC	140335	101744	143906
873	MC	89821	105828	125929
878	MC	90889	128528	163059
886	HC	39171	80944	124165
887	MC	64082	102365	162314
889	AD	51629	83484	124480
891	AD	39054	80899	124156
896	HC	56031	80437	128551
906	MC	66569	104507	162498
908	MNC	62589	121108	125169
912	MNC	40000	88122	128556
914	MNC	124879	91674	136716
916	AD	31533	89743	124083
919	MNC	65689	87132	134516
920	HC	42481	83076	124246
921	MNC	49510	80397	124778
922	MC	42493	83085	124499
923	HC	42509	82313	162091
925	MNC	67266	85641	129646
926	HC	31547	82180	125020
932	MNC	118715	83359	130054
934	HC	34734	80243	125126
938	AD	40980	81312	125155
941	MC	34747	82342	125038
945	MNC	36999	95633	130059

950	MNC	97200	121090	147503
951	HC	64170	85524	129340
952	MC	62224	106494	162991
961	MNC	59601	83003	129241
963	HC	89417	82195	129277
967	HC	55044	121099	147521
972	HC	45933	103629	130118
979	AD	39859	92217	132433
981	HC	52776	83316	129174
984	HC	33476	88036	134682
985	HC	62715	81405	125178
991	AD	40794	88024	149771
994	MNC	45943	91224	130128
997	MC	66630	121831	176861
1002	HC	65220	87265	139311
1004	MC	95705	94935	85689
1007	MC	88443	128538	166966
1010	MC	90566	136742	166921
1016	HC	42772	86245	133901
1018	AD	40817	87204	132197
1023	HC	65104	98811	139416
1028	MNC	40799	85925	132247
1031	MNC	40019	85654	129664
1032	MNC	63632	87319	134931
1033	MC	118718	118749	92291
1034	MNC	47953	86447	129585

1035	HC	51165	83364	133937
1041	AD	43071	86261	134940
1045	MNC	47210	83453	129304
1046	MNC	46396	83012	139042
1054	MC	62234	86832	132415
1056	AD	60741	92182	136737
1070	MC	60478	121072	165275
1074	MNC	53395	91404	141167
1078	MNC	79838	88559	142061
1080	MNC	35591	94387	132234
1081	AD	47168	91679	137533
1082	AD	47204	86236	132368
1086	HC	42785	92354	132388
1097	MNC	59610	86643	132261
1098	HC	42832	86698	132397
1106	MNC	49910	92696	130153
1109	AD	62695	91483	121756
1114	MNC	63489	92577	147530
1118	MNC	49768	98829	134885
1121	MC	90546	121747	166857
1122	MNC	52799	96010	136698
1123	HC	58043	97134	142098
1126	MC	128366	87086	138025
1130	MC	73037	122662	205570
1131	MNC	62943	91824	142150
1133	HC	118741	96218	136099

1140	MNC	63637	94649	138121
1144	AD	102040	87274	135299
1155	MNC	40845	91395	140722
1157	AD	66158	128789	160876
1164	AD	67223	91057	136220
1165	MNC	118729	94401	135583
1169	HC	119118	96300	141216
1170	AD	89957	92079	149650
1171	AD	63406	91640	137237
1182	MNC	50566	97164	142159
1183	MNC	66167	92273	160885
1186	MNC	62656	91441	143520
1187	MNC	48959	91954	142434
1190	HC	46417	89612	138005
1197	HC	66462	108336	142493
1206	HC	59981	96025	140777
1213	MC	47223	94453	135240
1215	MNC	60746	92296	136202
1217	MC	62984	94588	137001
1221	AD	48976	91967	136241
1222	HC	60003	96047	140791
1227	MNC	63838	99279	147855
1232	HC	59711	91905	136230
1246	MNC	69733	104531	142452
1247	MC	48857	91669	143169
1249	HC	59647	99187	135806

1250	HC	62240	91851	143901
1251	HC	62282	97152	143741
1254	AD	47228	91684	139062
1260	MNC	65239	97125	142068
1261	HC	62377	109394	139510
1262	AD	62433	103322	143676
1267	HC	80718	101747	160035
1268	MNC	64037	109403	143103
1269	MNC	68545	96150	160680
1271	MC	101965	143843	172384
1279	MNC	54757	99077	143694
1280	HC	60056	138890	141149
1281	AD	54780	102012	139224
1282	MC	62636	94397	143501
1283	AD	54785	101691	139235
1285	AD	51588	101710	139246
1288	HC	60664	97027	143597
1292	MC	118746	118760	103296
1296	AD	58022	97143	143732
1299	MC	80285	122150	204099
1300	MNC	119334	102132	143851
1301	HC	63793	102827	142459
1307	AD	63415	96266	141190
1308	AD	54752	102378	139266
1309	MNC	51605	98795	139278
1311	MC	97327	112538	143861

1314	MNC	63196	101774	160059
1318	MNC	60760	105915	149562
1321	MNC	118745	138909	143491
1326	MC	79318	217710	149483
1339	AD	56319	104363	149749
1341	AD	60417	103336	147406
1346	MNC	62299	105360	143750
1351	MNC	59615	97046	143667
1352	MNC	62319	104380	149814
1368	AD	65248	102387	143723
1371	AD	62998	109122	143792
1373	AD	63008	103707	143799
1379	AD	63014	109131	143815
1382	AD	66310	105481	149834
1384	MNC	64907	103285	149798
1385	AD	47574	101549	149553
1387	MC	78894	121785	141204
1389	MC	83147	129071	204791
1394	MC	68082	108348	171362
1398	MC	63227	95667	111278
1414	MNC	74557	121712	163393
1418	MNC	73642	121653	162557
1419	MNC	73656	121684	162969
